# Population genomics informs the management of harvested snappers across north-western Australia

**DOI:** 10.1038/s41598-024-77424-4

**Published:** 2024-11-04

**Authors:** Samuel D. Payet, Jim Underwood, Oliver Berry, Thor Saunders, Michael J. Travers, Corey B. Wakefield, Karen Miller, Stephen J. Newman

**Affiliations:** 1 Western Australian Fisheries and Marine Research Laboratories, Department of Primary Industries and Regional Development, Government of Western Australia, 39 Northside Drive, Hillarys, Western Australia 6025 Australia; 2 Australian Institute of Marine Science, Indian Ocean Marine Research Centre, Level 3, The University of Western Australia, Fairway, Crawley, WA 6009 Australia; 3 Department of Primary Industry and Resources, Northern Territory Government, 33 Vaughan St, Berrimah, NT 0828 Australia; 4 CSIRO Environomics Future Science Platform, The University of Western Australia, Crawley, WA 6009 Australia

**Keywords:** Genetics, SNP, Fishery, Stock structure, Stock identification, Animal migration, Conservation biology, Ecological genetics, Ecosystem services, Molecular ecology, Population dynamics

## Abstract

Failure to consider population structure when managing harvested fishes increases the risk of stock depletion, yet empirical estimates of population structure are often lacking for important fishery species. In this study, we characterise genetic variation in single nucleotide polymorphisms (SNPs) to assess population structure for three harvested species of tropical snappers across the broad (up to 300 km wide) and extensive (~ 4000 km) continental shelf of north-western Australia. Comparisons across ~ 300 individuals per species, showed remarkably similar patterns of genetic structure among *Lutjanus sebae* (red emperor), *L. malabaricus* (saddletail snapper) and *Pristipomoides multidens* (goldband snapper) despite subtle differences in biological and ecological traits. Low levels of genetic subdivision were reflected in an isolation by distance relationship where genetic connectivity increased with geographic proximity. This indicates extensive but not unlimited dispersal across the north-western Australian shelf. Our findings provide evidence of connectivity between current management areas, violating the assumption of multiple independent stocks. Spatial stock assessment models may be more suitable for the management of these species however demographic connectivity rates cannot be accurately estimated from the conventional population genetic approaches applied in this study. We recommend that managers aim to maintain adequate spawning biomass across current management areas, and assess stocks at finer scales, where practical.

**Supplementary Information:**

The online version contains supplementary material available at 10.1038/s41598-024-77424-4.

## Introduction

Population connectivity refers to the movement or dispersal of conspecific individuals across geographic space and is an important determinant of population dynamics^1^. For marine species, movement can occur throughout an individual’s lifetime, although in most teleost fishes it occurs primarily during the pelagic egg and larval phase^1^. Because pelagic larvae are cryptic and difficult to observe directly, numerous proxies are used to infer dispersal (reviewed by^2^). In fisheries management, estimates of population connectivity are used to inform the spatial scale at which stocks are assessed, *i.e.*, stock structure^3^. Failure to consider stock structure where it is present increases the risk of fishery depletion because it violates the unit stock assumption, which states that fish move freely within the stock, vital rates are homogenous (*i.e.*, growth, maturity, natural mortality, fishing mortality), and there is no immigration or emigration^4^. Despite this, population connectivity is often not included in fishery assessments due to a lack of empirical data, sometimes contributing to the decline and subsequent collapse of fish stocks^2,5,6^.

Because most marine fishes have broad geographic ranges and large population sizes, direct measurements of dispersal (*e.g.*, mark recapture^7^; telemetry^8^, or parentage analyses^9^) have been used less frequently in favour of more scalable, indirect approaches. For instance, comparisons of otolith chemistry (*e.g.*, ^10^), otolith shape (*e.g.*, ^11^) or parasite composition of host species (*e.g.*, ^12^) can indicate residency in adults or the ontogeny of movement across different environments following settlement (*e.g.*, ^13^). However, if there is limited spatial variation in seawater chemistry^14,15^ or if individuals are relatively site-attached after settlement (as per many reef-associated fishes; ^1^), then these approaches may not capture the life history stage where dispersal is greatest (*i.e.*, the pelagic egg and larval phase). The chemical composition of the otolith-core can indicate if alternate natal sources of larvae or juveniles exist within an area^16–18^ and if an understanding of the geography of seawater chemistry can be deduced from adults or via ground truthing (*e.g.*, ^19^) then individuals can be assigned to source locations. However, such comparisons may require cohort structured collections to account for the influence of environmental seasonality (*e.g.*, temperature and salinity variation) on larval otolith chemistry ^14,15^.

Population genetic comparisons are also commonly used to infer stock structure in fishes. These techniques compare the genetic composition (*i.e.*, DNA) of individuals across geographic space and can provide insights into dispersal that occurs during the cryptic larval phase. Numerous molecular techniques have been developed, with early studies comparing variation in allozyme markers, mitochondrial or nuclear markers (*e.g.,* ^20^) and microsatellite markers (*e.g.*, ^21^). More recently, methods that reduce genomes into a subset (typically, thousands) of mostly random genetic markers (*i.e.,* single nucleotide polymorphisms; SNPs) have become commonplace in population structure studies (e.g., ddRAD^22^ and DArTseq^23^) because they offer improved power to detect demographically independent stocks. Studies applying these techniques in harvested fishes have identified inter-annual variation in genetic structure (*e.g.*, ^24^), subtle genetic structure among populations separated by 10’s to 100’s of km’s^25^ or strict patterns of isolation well within the dispersal capabilities of the species^26^. These insights have led to renewed efforts to revisit stock structure in harvested fishes^6^ by integrating modern genetic techniques with multiple lines of evidence (e.g., otolith chemistry and parasite comparisons^4^).

**Fig. 1:**
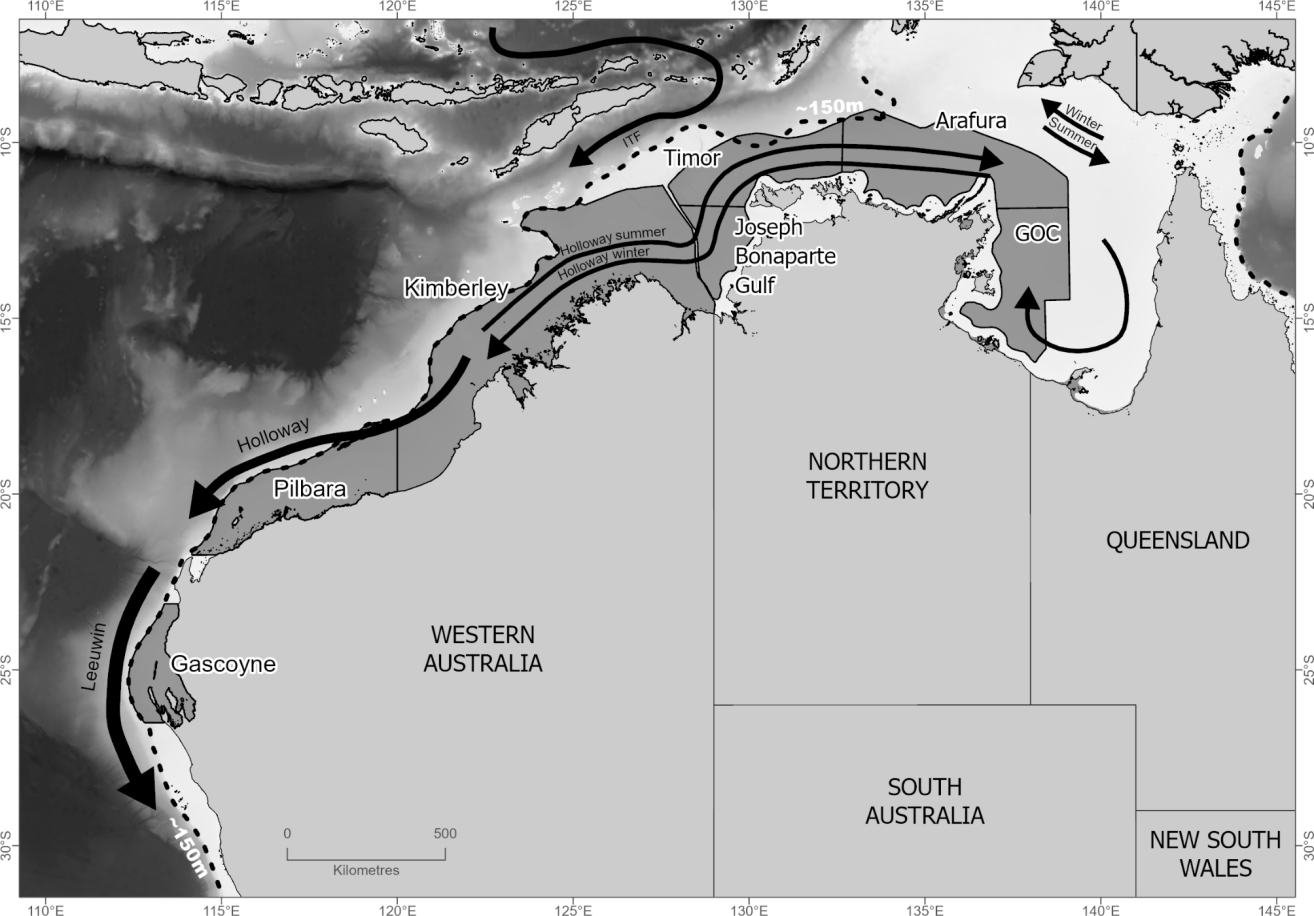
Fishery management areas in north-western Australia indicating geographic boundaries applied for the purpose of spatially aggregated stock assessments, except for *Lutjanus malabaricus* in the Timor and Arafura management areas which are assessed as a single stock^32^. Arrows indicate the major oceanographic features likely to influence larval dispersal including summer and winter variations of the Holloway Current. Widths of the arrows indicate the relative strength of water movement (redrawn from^43–46^). GOC = Gulf of Carpentaria; ITF = Indonesian Throughflow.

The continental shelf of northern and north-western Australia (collectively referred to as north-western Australia; NWA) provides a relatively broad (up to 300 km wide) and continuous (~ 4000 km long) band of habitat that extends from the western Gulf of Carpentaria through to the mid-west coast of Australia. The tropical snappers (Lutjanidae) are one of three principal family groups (see also, emperors; Lethrinidae and groupers, Epinephelidae) harvested by all fishing sectors in the area (commercial, charter, recreational, and customary) with the most significant catch by weight and gross value comprising the relatively slow growing and long lived *Lutjanus sebae* (red emperor), *L. malabaricus* (saddletail snapper) and *Pristipomoides multidens* (goldband snapper). All three species have Indo-Pacific distributions and are ubiquitous on the NWA shelf, though geographic variation in total catch across NWA is evident and likely reflects variation in habitat and depth preferences of each species (https://fish.gov.au/reports/species). The status of populations are determined using spatially aggregated stock assessment models in the seven management areas, which are for the most part, consistent among species (summarised in Fig. 1). Population structure has been investigated using a variety of genetic techniques that have identified low levels of genetic subdivision across NWA ^20,27–30^, but see^31^. Management (or assessment) units for these species are defined based on differences in otolith chemistry and parasite composition^32–34^, whilst also considering geopolitical boundaries, variation in habitat, biomass, fishing pressure and fishing methods^32,35^.

Recent studies applying reduced representation genotyping in fishes across NWA have identified species-specific patterns of population genetic structure that are inconsistent with management boundaries (*e.g.*, ^12,25,36,37^), indicating a need to revisit stock structure in the principal tropical snappers. Patterns of genetic subdivision among NWA fishes have been associated with variations in oceanographic currents, tidal regimes, geographic barriers, and bioregional areas (*e.g.*, ^12,21,25,37,38^). However, these studies focus on species that occupy the environmentally heterogenous coastal zone. It is not known if similar patterns are evident in the three snapper species that reside in the broad continental shelf, where habitat is relatively continuous and obvious barriers to dispersal are scarce. That said, the Holloway Current, Indonesian Throughflow, Leeuwin Current, and the extreme tidal range in parts of NWA have a differential influence on water movement across NWA (summarised in Fig. 1), and thus may influence larval dispersal and subsequent population connectivity.

*Lutjanus sebae* and *P. multidens* are also nominated indicator species in Western Australia where their stock status is used to infer the status of “like” species in the same fisheries resource^35^. The indicator species are nominated based on their vulnerability, biology, and socio-economic value, offering benefits in multi-species fisheries where it is impractical to monitor all harvested fishes^35^. Given that patterns of genetic structure can vary among co-distributed and even congeneric fishes^36,39^, it is necessary to consider whether patterns of connectivity in indicator species are representative of non-indicator species. Comparisons of genetic structure among closely related and co-distributed fishes will improve our understanding of those aspects of their biology or ecology that exert an influence on population connectivity (e.g., spawning behaviour^39,40^, pelagic larval duration^41^, larval swimming speed and behaviour^42^).

The primary aim of this study was to determine patterns of population genetic structure in *L. sebae*, *L. malabaricus*, and *P. multidens* across NWA, and provide advice for defining stock boundaries in these harvested fishes. Our results will provide insight into the factors that influence genetic structure in species occupying the broad and relatively continuous continental shelf habitat of NWA and indicate whether patterns of subdivision vary among closely related and co-distributed taxa.

## Methods

### Sampling

**Fig. 2:**
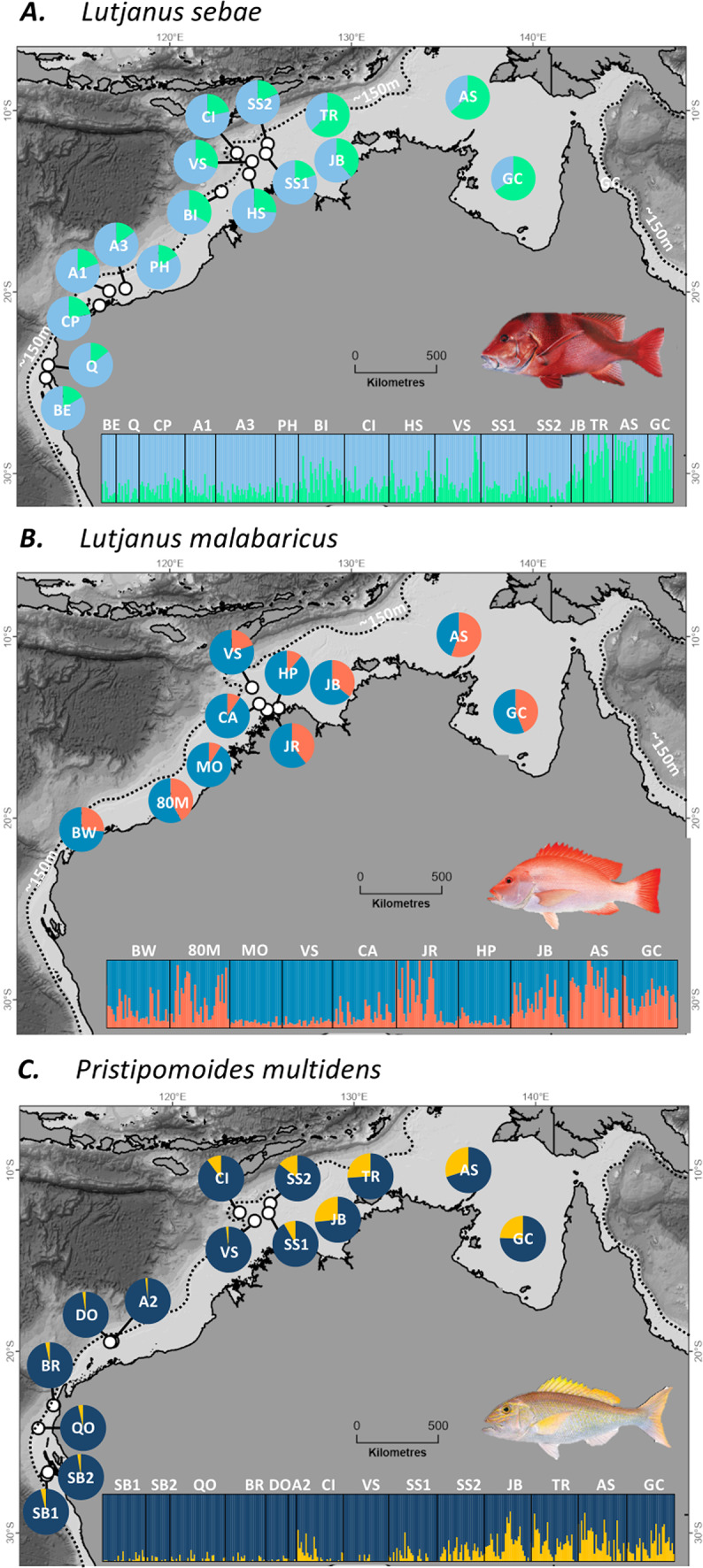
Individual admixture co-efficient estimates arranged by sampling sites for (**A**) *Lutjanus sebae*, (**B**) *L. malabaricus*, and (**C**) *Pristipomoides multidens*. Each vertical bar represents an individual and the colour indicates the admixture proportion at *K* putative populations. *K* values displayed here are based on the optimum cross-entropy criterion except for *L. sebae* where *K* = 2 (instead of *K* = 1; Fig. S2) is presented for the purpose of visualising genetic structure. Fish Illustrations reproduced with permission from www.anima.net.au, credit R. Swainston.

This study was performed in accordance with relevant guidelines and regulations (Department of Primary Industries and Regional Development exemption no. 251172823, see Additional Information for ethics declaration) and all methods are reported in accordance with ARRIVE guidelines. Tissue samples of *L. sebae* (n = 463), *L. malabaricus* (n = 373), and *P. multidens* (n = 462) were collected from multiple sites across each of the seven management areas in NWA extending to the southern edge of the distribution of each species (Figs. 1, 2; Table 1). Sampling occurred on research and commercial fishing vessels between 2012 and 2018 using hook-and-line or fish traps. Fish were euthanised via brain-spike and either processed immediately or stored frozen and processed at a later date. Fin or muscle tissue were taken from each individual and preserved in 98% ethanol.

**Table 1:** Genetic diversity estimates and sample sizes for each site including management areas and state jurisdictions for the three snapper species. *n* = sample size, *H*_o_ = observed heterozygosity, *H*_e_ = expected heterozygosity, *F*_IS_ = inbreeding coefficient. Parentheses; WA = Western Australia, NT = Northern Territory. Metadata associated with each site is shown in Table S1.

Species	Management area	Site	*n*	*H*o	*H*e	*F*_IS_
*Lutjanus sebae*	Gascoyne (WA)	BE	10	0.254	0.247	0.022
		Q	15	0.257	0.252	0.014
	Pilbara (WA)	CP	30	0.275	0.263	-0.025
		A1	20	0.281	0.262	-0.045
		A3	39	0.255	0.255	0.013
		PH	15	0.280	0.260	-0.042
	Kimberley (WA)	BI	30	0.263	0.259	-0.001
		CI	29	0.264	0.258	-0.004
		HS	30	0.258	0.256	0.010
		VS	30	0.262	0.256	-0.007
		SS1	30	0.255	0.253	0.009
		SS2	29	0.259	0.256	0.003
	Joseph Bonaparte Gulf (NT)	JB	8	0.268	0.249	-0.010
	Timor (NT)	TR	19	0.295	0.266	-0.080
	Arafura (NT)	AS	23	0.295	0.268	-0.075
	Gulf of Carpentaria (NT)	GC	17	0.294	0.266	-0.075
*Lutjanus malabaricus*	Pilbara (WA)	BW	33	0.209	0.199	-0.033
		80 M	31	0.253	0.219	-0.136
	Kimberley (WA)	MO	27	0.152	0.171	0.126
		CA	26	0.154	0.170	0.110
		VS	33	0.187	0.189	0.024
		JR	32	0.242	0.219	-0.087
		HP	27	0.160	0.174	0.099
	Joseph Bonaparte Gulf (NT)	JB	30	0.235	0.207	-0.114
	Arafura Sea (NT)	AS	28	0.286	0.237	-0.184
	Gulf of Carpentaria (NT)	GC	29	0.256	0.219	-0.147
*Pristipomoides multidens*	West Coast (WA)	SB1	27	0.147	0.163	0.117
		SB2	15	0.169	0.171	0.047
	Gascoyne (WA)	QO	34	0.187	0.182	-0.008
		BR	25	0.199	0.187	-0.040
	Pilbara (WA)	DO	14	0.186	0.183	0.023
		A2	5	0.175	0.163	0.041
	Kimberley (WA)	CI	29	0.162	0.176	0.095
		VS	28	0.159	0.167	0.065
		SS1	30	0.161	0.170	0.071
		SS2	29	0.171	0.177	0.049
	Joseph Bonaparte Gulf (NT)	JB	29	0.231	0.205	-0.107
	Timor Reef (NT)	TR	29	0.217	0.200	-0.070
	Arafura Sea (NT)	AS	30	0.231	0.210	-0.082
	Gulf of Carpentaria (NT)	GC	30	0.214	0.199	-0.054

### Reduced representation genotyping

Genomic DNA was extracted from either fin clips or muscle tissue samples using a salting out protocol modified from^47^ and purified with Zymo Plate filter plates (Zymo-Spin I-96). SNP discovery was conducted by Diversity Arrays Technology (DArT) which uses proprietary DArTseq technology to prepare reduced representation libraries for next generation sequencing^23,48^. DNA was digested using two restriction enzymes (*Pst*I-*Sph*I and *Pst*I-*Nph*I) and PCR consisted of denaturation (94 °C for 1 min) followed by 30 cycles of the following: 94 °C for 20 s, 58 °C for 30 s, 72 °C for 45 s, and then 72 °C for 7 min. PCR products were then pooled and standardised before being applied to a Illumina cBot bridge PCR system. Single end reads (77 bp) were sequenced on an Illiumina HiSeq 2500.

### Marker discovery and SNP quality control

Read assembly and initial quality control and SNP calling were conducted in the DArTsoft14 pipeline. Consistency in marker scoring (*i.e.*, reproducibility) was estimated by running technical sample replicates for 30% of the total number of samples. Sequences were blasted on GenBank to check for contamination and individuals containing sequences with even moderate E-values (< 1E^-06^) to any known bacterial genomes were removed from downstream analysis.

Each SNP dataset was subject to further quality filtering using the *R* v 4.2.3 packages^49^ *dartR* v 2.7.2^50^ and *Radiator* v 2.1.2^51^, applying similar filtering methods to all three species. First, we used *dartR* to retain one random SNP per read, markers with coverage between 5 and 50 X, markers with reproducibility greater than 95%, and markers with no less than 10% missing data. To account for potential sequencing errors, we then removed markers with a minor allele frequency less than 0.05 and markers that were in Hardy–Weinberg disequilibrium in any site using a 0.05 corrected mid *p*-value threshold^52^. After initial marker quality control, we then excluded any individuals with more than 20% missing data before using the *detect_mixed_genomes* function in *Radiator* to further screen for contaminated samples. Using this function, we excluded any individuals with heterozygosity estimates that exceed the outlier threshold within each site. Last, we used *OutFLANK* v0.1^53^ to identify outlier markers that may be affected by strongly divergent selection, using a 5% left and right trim for the null distribution of *F*_ST_, a minimum heterozygosity for loci of 0.1, and a 5% false discovery rate (*q*-value). The resulting datasets contained putatively neutral genetic markers for the investigation of population genetic structure. A summary of filtering parameters and the number of markers or individuals retained at each quality control step are shown in Table S2.

As part of the quality control process, we also investigated the influence of filtering thresholds on population genetic structure by filtering additional datasets without screening for missing data, Hardy–Weinberg disequilibrium, or minor allele frequency. Discriminant analyses of principal components (DAPC) in *Adegenet* v 2.1.4^54^ was conducted on each dataset to compare patterns of genetic clustering between datasets. To avoid overfitting the data, the number of discriminant functions and principal components were set based on *N*-populations – 1^55^.

### Population genetic structure

For each sampling site we calculated observed heterozygosity (*H*_O_), expected heterozygosity (*H*_E_), and fixation index (*F*_IS_) in *dartR*. To test for overall (global) genetic structure we conducted an AMOVA in the *R* package *strataG*^56^. To test for population structure between sites we estimated population-pairwise *F*_*ST*_^57^ also using the *R* package, *strataG*^56^. Significance was tested based on 1000 bootstrap replicates using *p* value thresholds corrected for multiple comparisons according to Benjamini & Yekuteili^52^. Sites with fewer than 15 samples were excluded from pairwise *F*_ST_ estimates due to small sample sizes.

Sparse Non-Negative Matrix Factorisation (sNMF) in the *R* package *LEA* v^58,59^ was used to estimate individual admixture coefficients. This approach provides similar outputs to the Bayesian STRUCTURE analyses^60^ but is robust to departures from traditional population genetic model assumptions^58^ that can lead to erroneous results where patterns of spatial autocorrelation (*i.e.*, isolation by distance, see below) are evident in the data^61,62^. By estimating individual admixture coefficients, we also provide an unbounded exploration of genetic structure in the data, including among individuals from sites that have small sample sizes (< 15).

Individual admixture coefficients were estimated at *K* = 1 to *K* = 10 putative populations with 20 replicate runs for each value of *K*. To determine the most likely number of populations (*K*) in our data we compared the cross-entropy criterion at each increase in *K*, and considered the optimum *K* as the value where the cross-entropy criterion initially decreases^58,59^.

## Isolation by distance

We investigated the relationship between genetic distance and geographic distance to determine if geographically close populations were more likely to experience genetic connectivity than distant populations (*i.e.*, isolation by distance; IBD). First, we performed a mantel test in the *R* package *dartR* to examine the relationship between linearised genetic distance (expressed as population pairwise *F*_ST_/(1-*F*_ST_)) and the shortest across-water distance (km, estimated in the *R* package *Marmap*; ^63^). Significance of mantel tests were determined based on 999 permutations and sites with fewer than 15 samples were excluded from the analyses.

We also conducted spatial auto-correlation analyses in *GenAlex* V6.5^64^. This approach estimates the correlation co-efficient (*r*) among individuals at increasing distance classes (km). In the presence of an IBD relationship, *r* should be positive among geographically proximal individuals and decline with increasing geographic distance. The point where *r* is no longer significantly different to zero indicates the “genetic patch size”, *i.e.*, the distance (km) where individuals are no longer significantly correlated^65^. For these analyses, we estimated multiple distance class correlograms due to uneven distances between sampling sites and tested the significance of *r* via 999 random permutations.

## Results

### Quality control

Initial DArT sequencing yielded approximately 20 to 35 thousand markers per species and after quality filtering in *R* we retained 3,074 markers across 379 *L. sebae* individuals, 2,277 markers across 296 *L. malabaricus* individuals, and 599 markers across 354 *P. multidens* individuals. Variation in marker discovery between species could reflect a combination of factors including differences in genome size, polymorphisms or missing data due to poor sample quality.

A total of 84 *L. sebae*, 60 *L. malabaricus*, and 90 *P. multidens* were excluded during the DArTsoft quality control pipeline based on evidence of bacterial contamination whilst five *L. sebae*, 17 *L. malabaricus*, and 18 *P. multidens* were excluded based on evidence of inter or intra-specific cross-contamination. This may reflect how the samples were processed following capture and highlight the importance of following protocols to minimise cross-contamination in the field, whilst also screening for contamination during quality control analyses.

Clustering analyses conducted on datasets that did not filter for minor allele frequency, Hardy–Weinberg equilibrium and call rate (missing data) yielded similar results for all three species (Fig. S1), except for one *L. malabaricus* site that clustered independently when SNPs were not filtered for missing data. This likely indicates lower quality genomic DNA from these individuals possibly due to how fish or samples were handled after capture.

### Broad scale genetic connectivity

Genetic structure amongst sample sites was significant but modest for all three species (AMOVA results: *L. sebae*, *F*_ST_ = 0.002, *p* < 0.001; *L. malabaricus*, *F*_ST_ = 0.013, P < 0.001; *P. multidens*,* F*_ST_ = 0.007, P < 0.001) and reflected relatively low genetic subdivision across the study area. Admixture coefficient estimates from sNMF provided further support for genetic connectivity among sites and did not reveal any spatially discrete genetic clusters (Fig. 2).

**Fig. 3:**
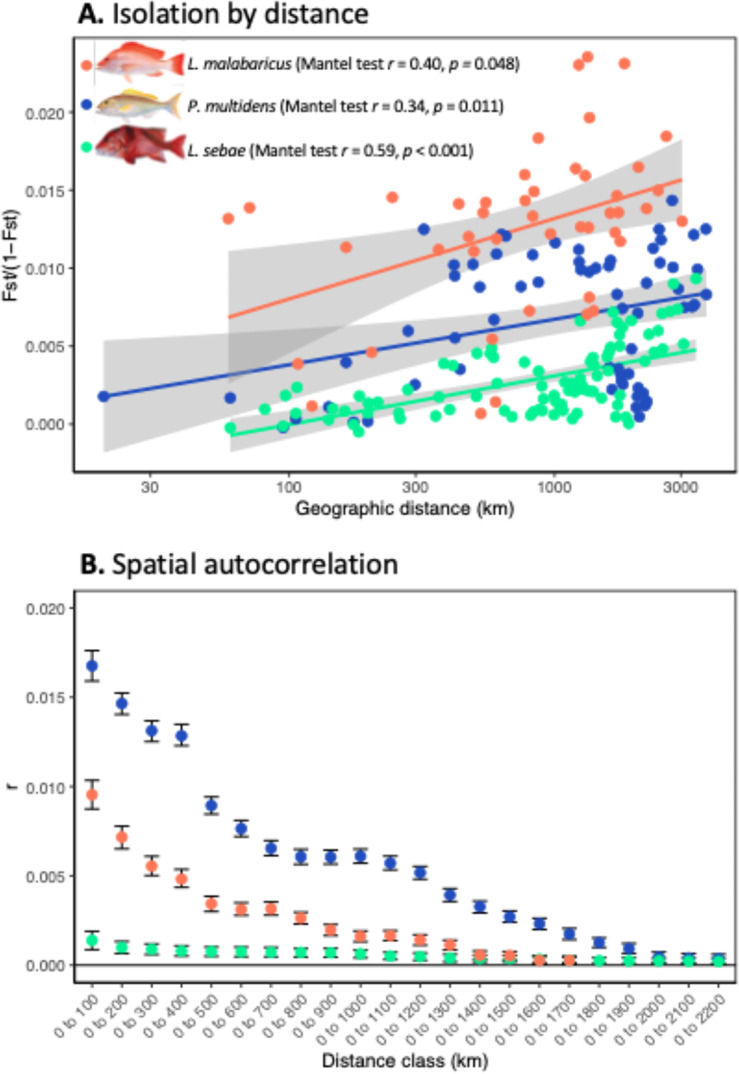
Relationships between geographic distance and genetic distance for each snapper species; (**A**) Isolation by distance plot from Mantel tests comparing population pairwise *F*_ST_ and coastal distance (km), and (**B**) spatial autocorrelation correlograms comparing the relationship (correlation co-efficient; *r*) between individual pairwise genetic distance at increasing distance classes (km). Error bars indicate 95% confidence intervals computed from 1000 bootstrap permutations.

Mantel tests indicated positive and significant IBD relationships between genetic distance (*F*_ST_) and across-water distance (km) for all three species (Fig. 3A), although *r*^2^ values were small (*L. sebae*, *r*^2^ = 0.345, *p* < 0.001; *L. malabaricus*, *r*^2^ = 0.161, *p* = 0.05; *P. multidens*,* r*^2^ = 0.115, *p* = 0.011). Spatial autocorrelation analyses also revealed a similar IBD relationship with positive autocorrelation (*r*) among geographically close individuals that declined at increasing distances classes (Fig. 3B). This indicates that the limit between gene flow and genetic drift is realised within the study area. Genetic patch size estimates were geographically large and concordant with the observed high levels of genetic connectivity among sites (*L. sebae* = 2200 km; *L. malabaricus* = 1700 km; *P. multidens* = 2200 km).

### Regional and interspecific genetic structure

**Fig. 4:**
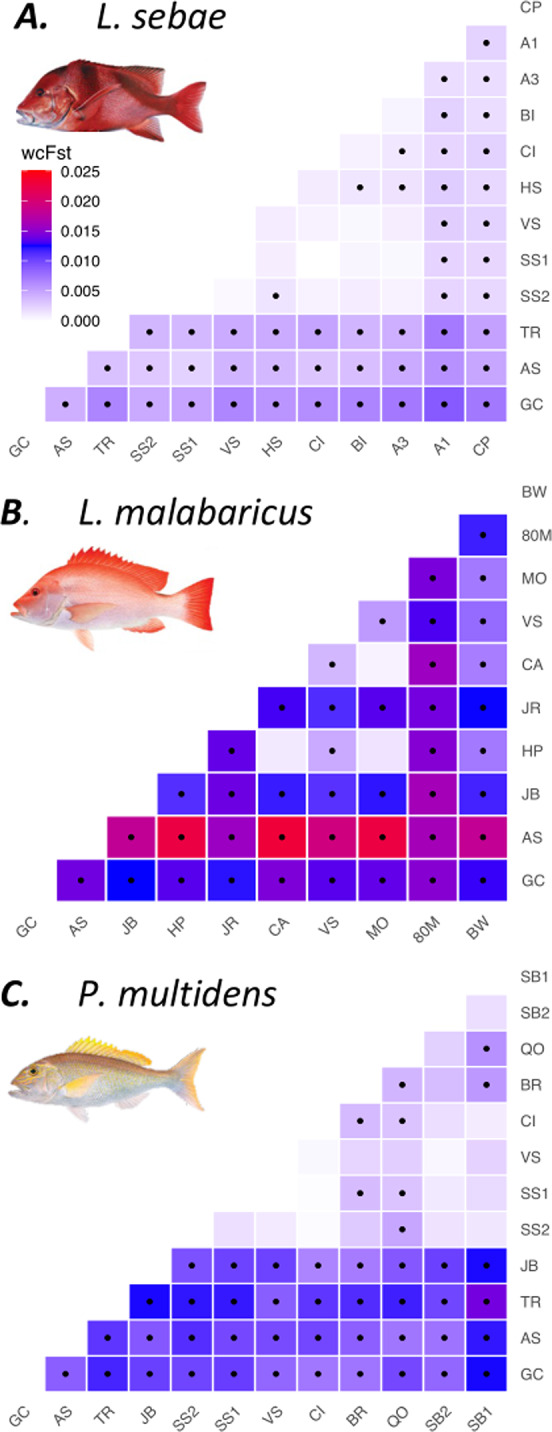
Pairwise *F*_ST_ comparisons among sites with 15 or more individuals for; (**A**) *Lutjanus sebae*; (**B**) *L. malabaricus* and; (**C**) *Pristipomoides multidens*. Black dots indicate pairwise *F*_ST_ comparisons that are significantly different from zero after correcting the *p*-value for multiple tests according to Benjamini & Yekuteili (52). wc*F*_ST_ = Weir & Cockerhams *F*_ST_^57^.

Amidst a background of genetic connectivity and IBD, we also identified subtle differences in structure within the study area. Pairwise *F*_ST_ comparisons revealed a pattern of higher relative connectivity within Western Australia and lower relative connectivity within the Northern Territory (Fig. 4). These regional differences were also supported by changes in genetic diversity, with Northern Territory sites displaying higher expected heterozygosity (*H*_e_) and greater inbreeding coefficient estimates (*F*_IS_) relative to most (but not all) Western Australian sites (Table 1).

Geographic patterns of genetic connectivity were consistent for all three species, except for three *L. malabaricus* sites (JR, 80M and BW) that exhibited greater genetic divergence that was not geographically well defined (Figs. 2, 3), and at higher *K* values showed evidence of clustering independently (Fig. S2). The magnitude of genetic structure also varied to some extent, with more modest pairwise *F*_ST_ comparisons in *L. sebae* than *P. multidens* and *L. malabaricus* (Fig. 4). This pattern was consistent with variation in genetic patch size estimates (Fig. 2B) and admixture coefficient estimates which identified *K* = 1 population as the most likely *K* for *L. sebae*, compared to *K* = 2 for *P. multidens* and *L. malabaricus* (Fig. S2).

## Discussion

In this study we used reduced representation genotyping to examine population genetic structure in three harvested finfish species in north-western Australia (NWA). Population genetic comparisons identified relatively high levels of genetic connectivity across ~ 4000 km of coastline, and an isolation-by-distance relationship amidst subtle geographic and interspecific differences.

Spatial comparisons primarily support high levels of genetic connectivity for all three species over their broad geographic range in NWA. This likely reflects the continuity of habitat along the continental shelf, the relatively wide shelf area that supports large population sizes thereby limiting the influence of genetic drift, and the absence of any strict geographic or oceanographic barriers to dispersal. In addition to this, we also identify an isolation by distance relationship (IBD), indicating that connectivity is greatest at local scales. In marine fishes with broad and relatively continuous distributions, patterns of IBD are common^67,68^, though in coastal fishes of NWA they are often simultaneously associated with genetic discontinuities across multiple physical barriers (*e.g.*, ^12,21,25,37,38^. For instance, a study on the coastally distributed congener *L. carponotatus,* identified genetic structure between the Kimberley and Canning bioregions and a genetic patch size that was far smaller than those estimated here (~ 300 km in the Kimberley versus 1700 to 2200 km in this study; ^25^). These results indicate that fishes occupying the continental shelf of NWA likely experience greater population connectivity than those in the coastal environments where geography and environment is more heterogenous.

Amidst a background of broad-scale genetic connectivity, the magnitude of structure also varied among neighbouring areas reflecting a pattern of higher relative connectivity in western Australia and lower relative connectivity in northern Australia. The geographic location of this genetic discontinuity is concordant with other studies on coastal species including *Lethrinus laticaudis* (grass emperor; ^21^), *Lates calcarifer* (barramundi; ^38^) and *Protonibea diacanthus* (black jewfish; ^12^). In this case, however, our data do not reflect a barrier between populations, but instead, greater connectivity in western Australia relative to adjoining northern Australia. Similar patterns are found among species where habitat differs between continous and patchy (e.g., coral grouper; ^26^ and marine snails; ^66^), although habitat patchiness does not increase in northern Australia^69^. Instead, this finding may support a greater influence of the dominant oceanographic features on genetic connectivity of snappers across the NWA shelf. In the Pilbara and Gascoyne regions, the Holloway and Leeuwin currents^43,44^ likely facilitate southward long-shelf connectivity as shown for other species in western Australia^70^. Indeed, during years when the Leeuwin Current is particularly strong, larvae of *L. malabaricus* and other tropical fishes have been obseved to disperse over 1000 km south of their natal populations^71^. In the Kimberley, the southward flowing Indonesian Throughflow appears to have a greater influence on connectivity than the comparatively weaker Holloway Current, which has a northward wind-driven aspect during the summer period^45,46^ when the three species are observed to spawn.

Spatial patterns of genetic diversity also varied with the northern Australia sites displaying higher heterozygosity estimates and lower inbreeding coefficient estimates than the majority of western Australia sites. Elliot^31^ proposed that variation in allozyme and mitochondrial DNA in *L. malabaricus* is consistent with colonisation of the northern Australia habitat from a refuge in western Australia that existed during periods of low sea level during the last glacial maxima (~ 6 kya). Our results could therefore reflect expansion of the tropical snappers into northern Australia followed by occasional connectivity and outbreeding with broader Indo-Pacific populations. Congruently, the northern Australia sites are adjacent to the Indonesian Archipelago and the east coast of Australia, where genetically distinct populations of each species not subject to this study exist^20,28,29,31^. Differences in diversity could also be due to sampling artefacts if a sample site comprises of groups of individuals subject to assortative mating (*i.e.*, Wahlund effects; ^72^). Ultimately, we emphasise caution when interpreting these differences given the magnitude of variation was relatively modest and not exclusive among areas (*i.e.*, some western Australia sites also displayed negative *F*_*I*S_ estimates). Broader sampling across each species’ Indo-Pacific distribution combined with demographic inference may provide further insights into the evolutionary histories of NWA populations.

Overall patterns of genetic structure and diversity were remarkably similar among the three species which may reflect shared aspects of their life history in NWA. For instance, the pelagic larval duration for *L. sebae* is approximately 40 days and is likely to be similar for the majority of lutjanids^73^. Seasonal comparisons of gonadosomatic indices and gonadal developmental stages of *L. sebae* and *L. malabaricus* indicate both species exhibit protracted spawning periods (*i.e.*, *ca*. 10 months) with bimodal peaks in reproduction during the austral spring and late summer/autumn (C. Wakefield unpub.). This suggests pelagic larvae are likely subject to similar broad oceanographic conditions during larval dispersal. This pattern is consistent with other demersal teleosts inhabiting the same locations with biannual peaks in reproduction related to photoperiod (e.g., ^74,75^). Although the spawning period of *P. multidens* exhibits a single, broad peak, it is also protracted (i.e., ca 8 months) over the same period (i.e., austral summer/autumn^76^, C. Wakefield unpub.).

Differences in spawning mode or spawning locations did not appear to influence genetic structure among species. *P. multidens* typically spawns in larger groups (with essential parity in sex ratios) concentrated along a depth range of approximately 80–120 m^77^. This contrasts with Ma et al.^39^, who found greater genetic connectivity among different grouper species that form larger aggregations (1000’s fish) versus smaller aggregations (100’s fish), although that study was conducted at a larger geographic scale (across ocean basins) and among more fragmented habitats than those studied here (*i.e.*, coral reefs). Together, our results indicate that extrinsic factors (oceanography and geography) are most likely the primary determinants of genetic connectivity in the three snapper species. Modest interspecific differences in the magnitude of *F*_ST_ and genetic patch size estimates may be due to unknown intrinsic factors, such as variation in effective population sizes (Ne), with slower rates of genetic drift in those with larger effective populations. Variation in demographic histories such as population bottlenecks, founder events or expansions could also result in spatio-temporal variation in Ne (*e.g.*, ^26^). Unfortunately, precise estimates of Ne can be challenging to obtain for marine fishes which typically have large, broadly dispersed and well-connected populations^78^.

*Lutjanus sebae*, *L. malabaricus* and *P. multidens* are used as indicator species to infer the status of other like species in respective management areas (*L. sebae* in the Pilbara and Kimberley management areas and *P. multidens* in the Kimberley and Gascoyne management areas). Patterns of population genetic structure were similar for the three snapper species, suggesting that the indicator approach applied across the species subject to this study is appropriate at least in terms of population genetic structure. Comparable studies should be conducted for emperor and grouper species that occupy the NWA shelf to determine if genetic structure is congruent in other principal family groups where the indicator species approach is applied^35^.

Our results show that the three snapper species comprise an interconnected meta-population with intra-population spatial heterogeneity in connectivity. This indicates that current management boundaries violate the unit stock assumption, as there is evidence of connectivity among stocks that are assessed and managed independently^3^. Under these population configurations, spatially structured stock assessments that incorporate estimates of connectivity among sub-populations may be more appropriate^68,79,80^. However, accurate estimates of demographic connectivity cannot be estimated using conventional population genetic approaches such as those applied here, nor would tag-recapture studies be appropriate because, like many reef fishes, the three snapper species are relatively site associated as adults^81^. Parentage (*e.g.*, ^82^) or kinship (*e.g.*, ^83^) studies may yield more precise estimates of dispersal by assigning juveniles to parents, though such studies would be impractical to apply at these scales given the broad distribution and presumably large population sizes of these species.

The approach by Spies et al.^68^ should also be considered, where stock boundaries do not exceed the median dispersal distance of each species. Median dispersal distances can be estimated from the IBD slope though this requires calculations of effective population size based on age-structured collections^84,85^, which would be difficult to obtain for the relatively long-lived snappers (e.g., ^35^). Whilst our estimates of “genetic patch size” indicate the spatial scale that populations are genetically alike, they do not necessarily represent demographic connectivity, as genetic panmixia between populations can occur when there are only a few migrants per generation (e.g., ^86^), and large population sizes may prevent genetic drift even if there is demographic isolation.

Until these practical constraints can be resolved, we follow the best practice principles for dealing with population structure in fishery assessments (reviewed by^2^). Given that adults are relatively sedentary, and dispersal primarily occurs during the larval phase, the current spatially aggregated assessments applied to each management area are considered appropriate^2^. The alternate magnitudes of structure within Western Australia and the Northern Territory jurisdictions support the separate management of stocks by each state entity. Within the Northern Territory, stocks are divided into areas defined based on otolith chemistry, gut parasite assemblages, and habitat configuration^32^. Given the absence of any discrete genetic clusters in this area, our results do not provide sufficient evidence to revise current management boundaries. Management areas in Western Australia are geographically larger than in the Northern Territory, higher relative genetic connectivity in Western Australia support these broader stock boundaries. However, we emphasise that the independent management of these areas should be applied with caution because declines in one stock may have broader implications for the population network, particularly if dispersal patterns are asymmetric. Thus, where practical, stocks should be assessed at finer spatial scales especially if there is evidence of variation in biological or ecological traits (*e.g.*, growth or carrying capacity). Finally, the assessment and maintenance of adequate adult spawning biomass across each species’ NWA distribution must be considered in a holistic sense, especially as connected populations are more resilient to disturbances and fishing pressure than fragmented populations^87^.

## Electronic supplementary material

Below is the link to the electronic supplementary material.

Supplementary Material 1

## Data Availability

Filtered and unfiltered genetic are available on Data Dryad repository 10.5061/dryad.j6q573npq.
