# Supplementary material for: Population genomics informs the management of harvested snappers across north-western Australia
Source: Sci Rep. 2024 Nov 4;14:26598. doi: 10.1038/s41598-024-77424-4 (PMC11535392; doi:10.1038/s41598-024-77424-4)
Supplement: Supplementary file 1 — Supplementary Material 1 [file 41598_2024_77424_MOESM1_ESM.docx]

## Supplementary materials

| Species | Site name | Site ID | n | Lat | Lon |
| --- | --- | --- | --- | --- | --- |
| *Lutjanus sebae* | Bernier Island | BE | 10 | -24.719 | 113.188 |
|  | Quobba | Q | 15 | -24.013 | 113.299 |
|  | Cape Preston | CP | 30 | -20.747 | 116.126 |
|  | Area 1 (PFTF) | A1 | 20 | -19.933 | 116.664 |
|  | Area 3 (PFTF) | A3 | 39 | -19.816 | 117.552 |
|  | Port Hedland | PH | 15 | -19.030 | 119.086 |
|  | Browse Island | BI | 30 | -14.484 | 122.845 |
|  | Cartier Island | CI | 29 | -12.348 | 123.699 |
|  | Heywood Shoal | HS | 30 | -13.492 | 124.345 |
|  | Vulcan Shoal | VS | 30 | -12.809 | 124.537 |
|  | Sahul Shelf 1 | SS1 | 30 | -12.367 | 125.287 |
|  | Sahul Shelf 2 | SS2 | 29 | -11.851 | 125.384 |
|  | Joseph Bonaparte Gulf | JB | 8 | -12.700 | 129.050 |
|  | Timor Reef | TR | 19 | -10.520 | 128.570 |
|  | Arafura Sea | AS | 23 | -9.310 | 136.240 |
|  | Gulf of Carpentaria | GC | 17 | -13.850 | 138.970 |
| *Lutjanus malabaricus* | Barrow Island | BW | 33 | -20.620 | 114.869 |
|  | Eighty Mile Offshore | 80M | 31 | -18.960 | 120.219 |
|  | Manari Offshore | MO | 27 | -17.233 | 121.627 |
|  | Cassini Island | CA | 26 | -13.701 | 124.933 |
|  | Vulcan Shoal | VS | 33 | -12.822 | 124.515 |
|  | Jamieson Reef | JR | 32 | -14.025 | 125.365 |
|  | Hat Point | HP | 27 | -13.937 | 125.987 |
|  | Joseph Bonaparte Gulf | JB | 30 | -12.700 | 129.050 |
|  | Arafura Sea | AS | 28 | -10.020 | 136.250 |
|  | Gulf of Carpentaria | GC | 29 | -14.210 | 138.900 |
| *Pristipomoides multidens* | Shark Bay 1 | SB1 | 27 | -26.650 | 113.140 |
|  | Shark Bay 2 | SB2 | 15 | -26.830 | 113.090 |
|  | Quobba Offshore | QO | 34 | -24.233 | 112.621 |
|  | Bruboodjoo | BR | 25 | -22.955 | 113.431 |
|  | Dampier Offshore | DO | 14 | -19.474 | 116.555 |
|  | Area 2 (PFTF) | A2 | 5 | -19.447 | 116.691 |
|  | Cartier Island | CI | 29 | -12.348 | 123.699 |
|  | Vulcan Shoal | VS | 28 | -12.809 | 124.537 |
|  | Sahul Shelf 1 | SS1 | 30 | -12.367 | 125.287 |
|  | Sahul Shelf 2 | SS2 | 29 | -11.851 | 125.384 |
|  | Joseph Bonaparte Gulf | JB | 29 | -12.700 | 129.050 |
|  | Timor Reef | TR | 29 | -10.400 | 130.820 |
|  | Arafura Sea | AS | 30 | -10.020 | 136.250 |
|  | Gulf of Carpentaria | GC | 30 | -13.850 | 138.970 |

**Table S1.** Metadata associated with each sample site including the site name, site ID, sample size (n), and geographic co-ordinates in decimal degrees format.

**Table S2.** Filtering parameters for each dataset and number of markers/individuals retained at each stage of filtering.

|  |  | *Lutjanus sebae* | | *Lutjanus malabaricus* | | *Pristipomoides multidens* | |
| --- | --- | --- | --- | --- | --- | --- | --- |
| filter | threshold | SNPs | individuals | SNPs | individuals | SNPs | individuals |
| Initial dataset from DArT |  | 20,709 | 379 | 34,278 | 313 | 28,753 | 372 |
| Secondaries (single SNP per read) |  | 17,609 | 379 | 26,900 | 313 | 25,744 | 372 |
| Read depth (coverage) | 5x to 50x | 14,297 | 379 | 24,419 | 313 | 17,373 | 372 |
| Reproducibility | 0.95 | 13,085 | 379 | 20,524 | 313 | 15,479 | 372 |
| Maximum missing data by locus | 0.1 | 11,515 | 379 | 14,142 | 313 | 5,607 | 372 |
| Minimum minor allele frequency (MAF) | 0.05 | 4,647 | 379 | 3,371 | 313 | 1,411 | 372 |
| Hardy-weinberg equilibrium (HWE) |  | 3,077 | 379 | 2,277 | 313 | 601 | 372 |
| Maximum missing data by individual | 0.2 | 3,077 | 377 | 2,277 | 309 | 601 | 362 |
| Mixed genomes (Radiator) | outliers | 3,077 | 374 | 2,277 | 296 | 601 | 354 |
| Outlier markers (OutFLANK) | 5% trim | **3,074** | **374** | **2,277** | **296** | **599** | **354** |

**Fig. S1** Scatterplots of discriminant analyses of principal components (DAPC) indicating population genetic structure inferred from SNP data for *Lutjanus sebae*, *L. malabaricus*, and *Pristipomoides multidens* when turning off key filtering parameters (HWE = Hardy-Weinberg Equilibrium, MAF = Minor allele frequency, Callrate = missing data by locus).

**Fig. S2** Individual admixture co-efficient estimates arranged by sampling sites for A. *Lutjanus sebae*, B. *L. malabaricus*, and C. *Pristipomoides multidens* at increasing values of *K* (left). Corresponding correlograms at *K* = 1 to *K* = 10 are shown for each species (right), indicating the most parsimonious *K* as denoted by black arrows.
